# Physiological and biochemical characterization of egg extract of black widow spiders to uncover molecular basis of egg toxicity

**DOI:** 10.1186/0717-6287-47-17

**Published:** 2014-05-16

**Authors:** Yizhong Yan, Jianjun Li, Yiya Zhang, Xiaozhen Peng, Tianyao Guo, Jirong Wang, Weijun Hu, Zhigui Duan, Xianchun Wang

**Affiliations:** Key Laboratory of Protein Chemistry and Developmental Biology of Educational Ministry of China, College of Life Sciences, Hunan Normal University, Changsha, 410081 P. R. China

**Keywords:** Black widow spider (*L. tredecimguttatus*), Protein composition, Egg, Molecular mechanism, Property analysis, Toxicity

## Abstract

**Background:**

Black widow spider (*L. tredecimguttatus*) has toxic components not only in the venomous glands, but also in other parts of the body and its eggs. It is biologically important to investigate the molecular basis of the egg toxicity.

**Results:**

In the present work, an aqueous extract was prepared from the eggs of the spider and characterized using multiple physiological and biochemical strategies. Gel electrophoresis and mass spectrometry demonstrated that the eggs are rich in high-molecular-mass proteins and the peptides below 5 kDa. The lyophilized extract of the eggs had a protein content of 34.22% and was shown to have a strong toxicity towards mammals and insects. When applied at a concentration of 0.25 mg/mL, the extract could completely block the neuromuscular transmission in mouse isolated phrenic nerve-hemidiaphragm preparations within 12.0 ± 1.5 min. Using whole-cell patch-clamp technique, the egg extract was demonstrated to be able to inhibit the voltage-activated Na^+^, K^+^ and Ca^2+^ currents in rat DRG neurons. In addition, the extract displayed activities of multiple hydrolases. Finally, the molecular basis of the egg toxicity was discussed.

**Conclusions:**

The eggs of black widow spiders are rich in proteinous compounds particularly the high-molecular-mass proteins with different types of biological activity The neurotoxic and other active compounds in the eggs are believed to play important roles in the eggs’ toxic actions.

## Background

Black widow spider (*L. tredecimguttatus*) is one of the medium-sized spiders and taxonomically belongs to Phylum Arthopoda, Arachnida, Araneae, Theridiidae, genus *Latrodectus*[1]. It is one of the most poisonous spiders in the world. The venom secreted by its venomous glands is a mixture of biologically active components which have diverse actions on prey as well as human victims. Many studies including our previous work analyzed the venom secreted by its venomous glands and described the biological properties and structures of some venomous proteins in the venom [2–6]. Interestingly, black widow spider, different from other poisonous animals, has toxic components not only in the venomous glands, but also in other parts of the body (such as legs and abdomen) and its eggs. For example, Sampayo [7] reported that the eggs of *L. mactans* have hemolytic properties, and that this action is evident on red blood cells of the rabbits, without any hemolytic action occurring following injection into guinea pigs, horses, and humans. He also mentioned that there were differences between the venom injected by the spider and the one contained in the eggs. Using chemical and physiopharmacological methods, Buffkin et al. [8] made a primary study on the purification of toxic components from spiderlings and the eggs as well as abdomen of adult spiders. They demonstrated that the toxins were contained in the eggs themselves and not in the egg shell, and the extracts of the eggs of *Loxosceles* and other several spider species did not display any toxicity to mice. When the extract of the eggs of black widow spider was fractionated on Sephadex G-50, three major peaks were obtained and most of the lethal property appeared in the first and largest peak. Akhunov et al. [9] demonstrated that the egg extract of black widow spiders contains toxins immunologically different from those of spider venom glands. Thus, it is biologically important to comprehensively investigate and compare the characteristics of the eggs and venom. Although there have been sporadic reports about the toxicity of the eggs of black widow spider, up to now there have been no reports on the systematic analysis of the eggs. In the present work, we made a systematical physiological and biochemical analysis of the aqueous extract of black widow spider eggs. The main characteristics of the egg extract were compared with those of the venom and the implications were discussed.

## Results

### Protein content and hydrolase activity of extract

Using Bradford method, the protein content of the lyophilized egg extract was determined to be 34.22% (Table 1). The determination results of activities of several hydrolases in the extract are listed in Table 1. It could be seen that the extract displayed certain activities of protease, alkaline phosphatase, acid phosphatase, acetylcholinesterase and hyalurinidase, suggesting that the eggs of black widow spiders are rich in hydrolases.

**Table 1:** **Partial properties of the egg extract**

Item	Determined value
Protein content	34.22%
Protease activity	0.332 U/mg
Alkaline phosphatase activity	0.005 U/mg
Acid phosphatase activity	0.007 U/mg
Acetylcholinesterase activity	0.004 U/mg
Hyaluronidase activity	3.765 U/mg
LD_50_ in mice	3.32 mg/kg

### Extract toxicity to animals

In order to determine whether the egg extract contained components toxic to animals, the extract sample was intra-abdominally injected into mice and cockroaches. The experiments showed that the egg extract contains the components toxic to the mice. Within 10 min after intraperitoneal injection of the egg extract in mice, the animals displayed a variety of poisoning symptoms such as chill, huddle and trembling, hair fold, shortness of breath, flaccid paralysis, difficulty to open eyes, urinary incontinence and hypothermia. As the time prolonged and the doses increased, the symptoms were aggravated and death occurred, whereas the mice injected with physiological saline acted normally. Intraperitoneal LD_50_ in mice of the extract was calculated to be 3.32 mg/kg body weight (Table 1). It was found that the egg extract is also toxic to *P. americana*. Intra-abdominal injection of the extract into cockroaches made the animals display a series of poisoning symptoms including sluggishness or paralysis, trembling and lags in response. These data suggested that the eggs of black widow spiders contain the active components toxic to both mammals and insects.

### Effect of extract on neuromuscular transmission

**Figure 1:**
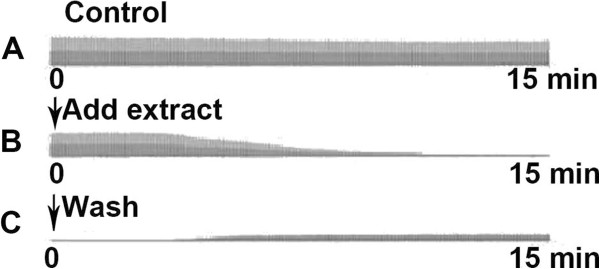
**Effect of egg extract on the neuromuscular transmission in mouse isolated phrenic nerve-hemidiaphragm preparations.** Legend: The neurotoxicity of the egg extract was demonstrated by detecting its effect on the amplitude of phrenic muscle contraction caused by the electrical stimulation of phrenic nerve. **(A)** control; **(B)** test, adding the egg extract at a concentration of 0.25 mg/mL after stable contraction in Tyrode’s solution for about 20 min; **(C)** washing the test preparation with Tyrode’s solution after blockade.

The electrophysiological experiments using mouse isolated phrenic nerve- hemidiaphragm preparations were performed to detect the effects of the egg extract on neuromuscular transmission. In the control experiments with the preparation immersed in Tyrode’s solution without adding egg extract, there were not obvious changes during 2–3 h in the amplitude of phrenic muscle contraction caused by the electrical stimulation of phrenic nerve. The representative trace is shown in Figure 1A. However, the addition of egg extract at a concentration of 0.25 mg/mL caused the contraction to be reduced gradually and abolished completely within 12.0 ± 1.5 min (n = 3), indicating that the neuromuscular transmission was blocked by the neurotoxic components in egg extract (Figure 1B). After blockade, repeated wash with fresh Tyrode’s solution led to partial recovery of the contraction (Figure 1C), suggesting that the action of some components in the egg extract was reversible. In addition, in order to acquire even more detailed information, the extract was separated with a 10 000-dalton ultrafilter into low-molecular-mass (<10 kDa) and high-molecular-mass (>10 kDa) fractions. The electrophysiological experiments demonstrated that both of the two fractions displayed inhibitory activity towards the nerve-evoked contraction of the phrenic muscle in mouse isolated phrenic nerve- hemidiaphragm preparation. However, the high-molecular-mass fraction exhibited much higher activity (the figures not shown), suggesting that the neurotoxicity of the eggs to the mammals was primarily due to the activity of the components with high molecular masses.

### Effects of egg extract on sodium channels in DRG neurons

**Figure 2:**
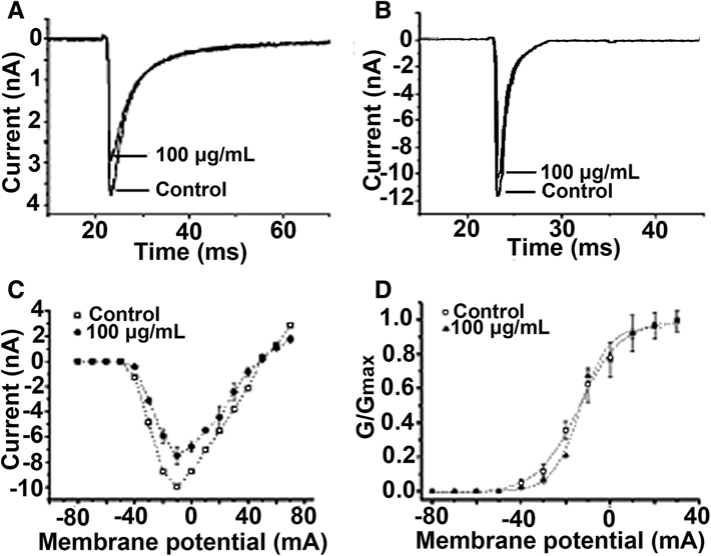
**Effects of egg extract on voltage-gated sodium channels in rat DRG neurons.** Legend: Patch-clamp technique was used to measure the sodium currents of rat DRG neurons (n = 5). **(A)** TTX-R Na^+^ currents were induced by a 50-ms step depolarization to −10 mV from a holding potential of −80 mV every 5 seconds in small DRG neurons in the presence and absence of 100 μg/mL egg extract. **(B)** TTX-S Na^+^ currents were induced by a 50-ms step depolarization to −10 mV from a holding potential of −80 mV every 5 seconds in large DRG neurons in the presence and absence of 100 μg/mL egg extract. **(C)** Current–voltage curves of TTX-R Na^+^ channels in the presence and absence of 100 μg/mL egg exact. **(D)** Steady-state activation curves of TTX-R Na^+^ channels in the presence and absence of 100 μg/mL egg extract. G was normalized to the peak membrane conductance (Gmax ) at +30 mV. The curve was fitted with the equation G = Gmax /[1 + exp (V0.5-V)/k].

After the two types of voltage-gated sodium channels expressed in DRG neurons were separated by addition of TTX to the external bath solution, the effects of egg extract on TTX-R and TTX-S sodium channels were investigated. The results (Figure 2) showed that addition of the egg extract could decrease the amplitude of sodium currents. The extract at a concentration of 100 μg/mL reduced the amplitudes of TTX-R and TTX-S sodium currents in DRG neurons by 14.36 ± 5.13% (Figure 2A, n = 5) and 12.21 ± 2.02% (Figure 2B, n = 5), respectively. After being depressed by the extract, the shapes of current traces were similar to those of controls, suggesting that the extract did not significantly affect the activation and inactivation kinetics of the voltage-gated sodium channels.

Current–voltage (I–V) curves of TTX-R Na^+^ channels in the absence and presence of the extract were prepared to probe into the effect of the extract on the opening of sodium channels. The resultant current–voltage (I–V) curves of the sodium channels before and after treatment with 100 μg/mL egg extract are shown in Figure 2C. It can be seen that, under control experimental conditions, TTX-R sodium currents were initially elicited at about −50 mV, reached maximal amplitude at about −10 mV, and reversed at about +50 mV. After treatment with 100 μg/mL egg extract, inhibition of the sodium currents was observed. However, the extract did not change the threshold of activation voltage, the activation voltage for the inward peak currents and the reversal voltage of the inward currents, suggesting that the interaction of the toxic components in the extract with the sodium channels did not alter the ion selectivity of the channels. Besides, the inspection of the conductance-voltage curves of the sodium channels in the absence and presence of 100 μg/mL egg extract found that that the extract did not lead to obvious changes in the channel conductance at voltages varying from −80 to +30 mV (Figure 2D), indicating that the extract caused no significant changes in the activation kinetics of the sodium channels in rat DRG neurons.

### Effects of egg extract on potassium channels in DRG neurons

**Figure 3:**
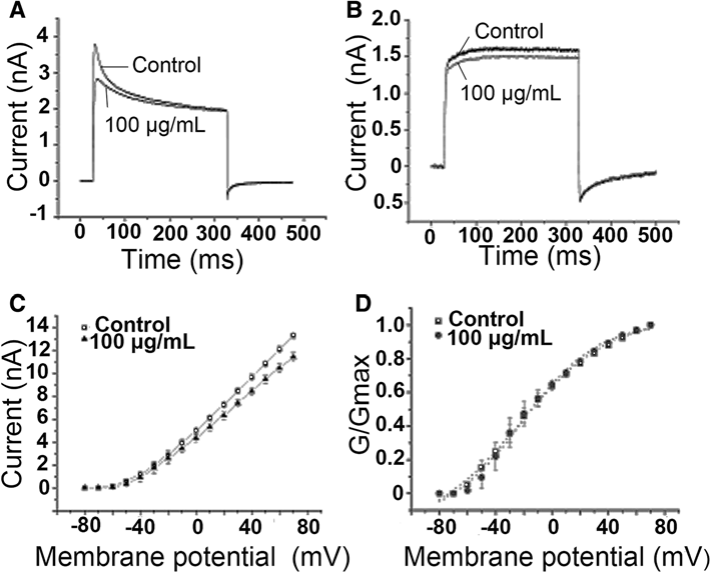
**Effects of the egg extract on voltage-gated potassium channels in DRG neurons.** Legend: Patch-clamp technique was used to measure the potassium currents of rat DRG neurons (n = 5). **(A)** 100 μg/mL egg extract inhibited about 13.96 ± 5.07% of the transient outward K^+^ currents. **(B)** 100 μg/mL egg extract had not obvious effect on the delayed rectifier K^+^ currents. **(C)** current–voltage curves of K^+^ currents in the presence and absence of 100 μg/mL egg extract. **(D)** steady-state activation curves of K^+^ channels in the presence and absence of 100 μg/mL egg exact.

To investigate whether the egg extract could influence potassium channels in DRG neurons, we used the patch-clamp technique to measure the whole-cell potassium currents of DRG neurons. The outward potassium currents were isolated by blocking TTX-S sodium currents with 0.3 mM TTX and by applying test pulses close to the sodium reversal potential to minimize the contribution of the remaining TTX-R sodium currents. Calcium currents and calcium-activated currents were eliminated by removing external calcium and by including EGTA in the patch pipette. As shown in Figure 3A, bath application of 100 μg/mL egg extract suppressed the potassium currents by 13.96 ± 5.07% (n = 5). However, the egg extract at the same concentration had not obvious effect on the delayed rectifier K^+^ currents (Figure 3B), suggesting that the eggs might contain no delayed rectifier K^+^ current-inhibiting components.

The current–voltage (I–V) curve of potassium channels (Figure 3C) shows that before and after the treatment with 100 μg/mL egg extract the activation voltage of potassium channels remained to be the same (about −50 mV), indicating that the active components in the extract did not affect the activation voltage of the channels. Figure 3D shows the conductance–voltage curves obtained before and after the extract treatment, which demonstrated that the treatment with the extract caused no obvious changes in the activation kinetics of K^+^ channels in rat DRG neurons.

### Effects of egg extract on calcium channels in DRG neurons

**Figure 4:**
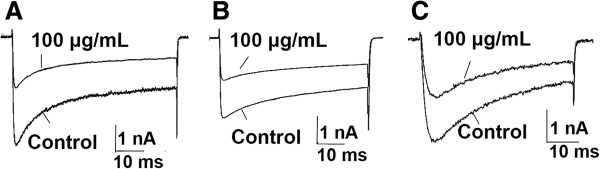
**Effects of the egg extract on voltage-gated calcium channels in DRG neurons.** Legend: Patch-clamp technique was used to measure the calcium currents of rat DRG neurons (n = 5). **(A)** 100 μg/mL extract inhibited 47.7 ± 1.9% of total Ca^2+^ currents. **(B)** 100 μg/mL extract inhibited 48.7 ± 5.8% of HVA-Ca^2+^ currents. **(C)** 100 μg/mL extract inhibited 43.5 ± 3.6% of LVA-Ca^2+^ currents.

The effects of egg extract on the total calcium currents in rat DRG neurons which were elicited by a 150-ms depolarization to 0 mV from a holding potential of −90 mV were first determined. The result showed that the treatment with 100 μg/mL egg extract inhibited 47.7 ± 1.9% of total calcium currents in rat DRG neurons (Figure 4A, n = 5). Then the effects of the extract on different types of calcium channels in DRG neurons were detected. The main voltage-gated calcium channels that the rat DRG neurons express can be divided into two groups: low-voltage-activated (LVA) calcium channels (T-type) and high-voltage-activated (HVA) channels (L-, N-, P-, Q- and R-type channels) that are activated by different depolarization voltages. LVA calcium channels can be activated by a 100-ms step depolarization to −50 mV from a holding potential of −90 mV, whereas only HVA currents are activated if the cells is depolarized from a holding potential of −40 mV to 0 mV [10–12]. Therefore, we employed different activation strategies to activate the cells to detect the effects of the egg extract on HVA- and LVA-Ca^2+^ channels, respectively. The results are shown in Figure 4. It can be seen that 100 μg/mL egg extract decreased the HVA and LVA calcium currents by 48.70 ± 5.76% (Figure 4B, n = 5) and 43.50 ± 3.64%, respectively (Figure 4C, n = 5), indicating that the spider eggs contain active components that inhibit the calcium channels in the DRG neurons.

### Mass spectrometric analysis

**Figure 5:**
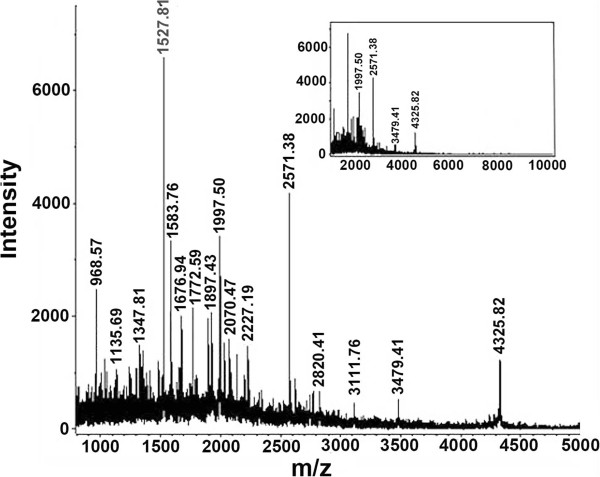
**MALDI-TOF mass spectrum.** Legend: MALDI-TOF mass spectrometric analysis of the low-molecular-mass fraction (<10 kDa) of egg extract. The inset shows the corresponding whole mass spectrum.

While the gel electrophoresis can efficiently separate and determine the large proteins, MALDI-TOF mass spectrometry can be used to accurately analyze the low-molecular-mass proteins and peptide components in the egg extract. The representative mass spectrum of the low-molecular-mass fraction (<10 kDa) is shown in Figure 5. The analytical results showed that the egg extract contained certain amounts of peptide components with molecular masses below 5 kDa, most of which were concentrated in the range of about 0.9 to 2.3 kDa. There were few peptides to be distributed between 5 kDa and 10 kDa that had been suppressed due to low abundance of the peptides in the sample (inset in Figure 5).

### Comparison of proteomes of egg extract and venom by SDS-PAGE

**Figure 6:**
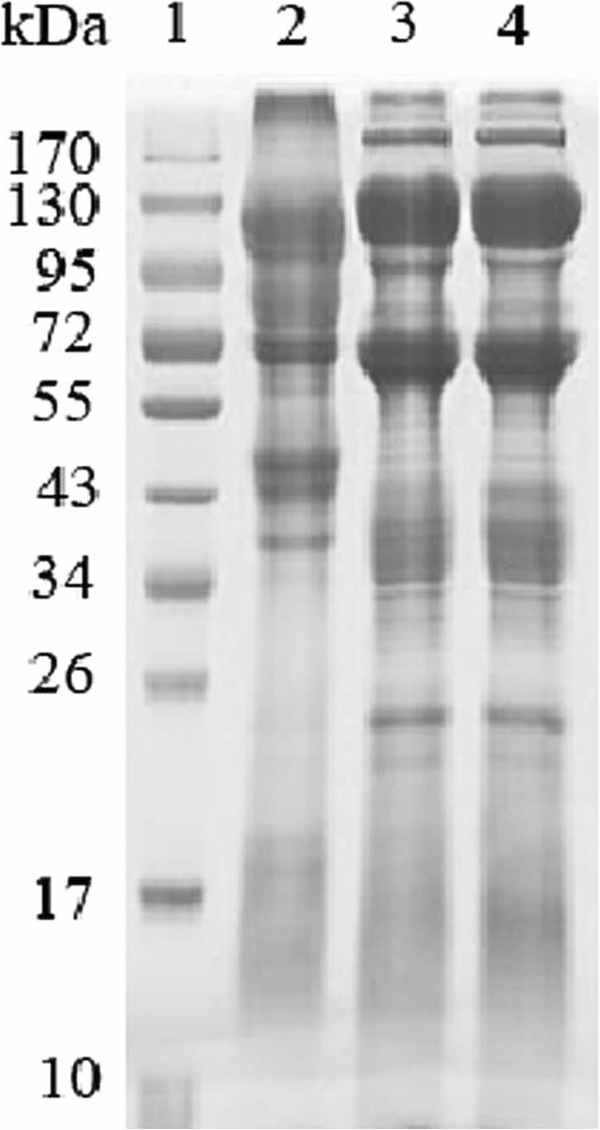
**1D SDS-PAGE image of the extract.** Legend: Comparative analysis of the egg extract and venom of black widow spider by SDS-PAGE. Lane 1, molecular mass marker; Lane 2, venom; Lane 3, egg extract prepared with ddH_2_O; Line 4, egg extract prepared with 1.0% sodium deoxycholate.

The SDS-PAGE images of the egg extract and venom of the black widow spider are shown in Figure 6. It can be found from the figure that the two samples had distinct protein distribution profiles. The protein composition of the egg extract was more complex than that of the venom. Most of proteins in the extract were distributed in the MW range from about 34 kDa to above 170 kDa, with the highest abundant protein bands at around 65 kDa and 130 kDa (lanes 3 and 4 in Figure 6), whereas the main protein components of the venom were distributed in the MW range from about 43 to 120 kDa (lane 2 in Figure 6). Compared with venom, egg extract had more proteins with molecular mass above 170 kDa and below 43 kDa. In addition, there were only few protein bands in the low-molecular-mass regions of the two sample lanes, suggesting that both eggs and venom were rich in high-molecular-mass proteins. In addition, we investigated the extractability of the proteins in eggs by comparing the extracts prepared from the eggs with or without the help of a detergent. The extract sample loaded in lane 4 was prepared by extraction with 1.0% sodium deoxycholate, a mild detergent. It was found that the protein file of the extract prepared with water (lane 3) was not significantly different from that of lane 4, indicating that the proteins in the eggs were highly water-soluble and could be efficiently extracted with a common aqueous buffer solution of weak ionic strength or water and the extract sample prepared in the present work had representativeness.

### 2D-PAGE of egg extract

**Figure 7:**
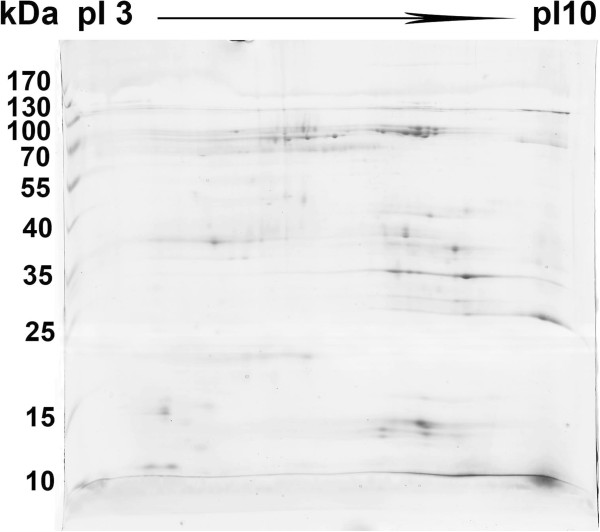
**2D-PAGE image of the egg extract.** Legend: 2D-PAGE analysis of the egg extract, showing the MW and pI distributions of the proteins in the eggs.

In order to further investigate the distribution of large-molecular-mass proteins in the eggs, besides SDS-PAGE, 2D-PAGE was also performed using a wide p*I* range IPGphor strip (pH 3-10 L).The representative 2D-PAGE image of the egg extract is shown in Figure 7. It can be seen that protein spots were numerous and roughly equally distributed in the gel, suggesting that the protein composition of the eggs was complex and the eggs contained a range of proteins varying in molecular masses and isoelectric points. In addition, it is worthy noting that in the range above about 100 kDa, there are only a few protein dots, suggesting that most of the proteins with high molecular masses were lost during their transferring from the immobilized pH gradient gel strip into polyacrylamide separation gel.

## Discussion

The experimental results demonstrated that the eggs of black widow spiders were rich in proteinous components. The protein content of the egg extract reached 34.22%, which was, however, lower than that (55.16%) of the venom secreted by the spider’s venomous glands [6], suggesting that the egg extract contains more non-proteinous components than the venom. Gel electrophoresis and mass spectrometric analyses showed that the eggs, like the venom [6], are composed mainly of large proteins and the content of low-molecular-mass proteins or peptides is relatively low.

After intra-abdominally injection of the egg extract in mice and cockroaches, the animals displayed a variety of poisoning symptoms. Moreover, the animals injected with a high dose of the extract died within three hours. These results suggested that the eggs of the spiders contained the components toxic to mammals and insects. Interestingly, our previous work made a global analysis of the protein composition of the spider eggs using a combined proteomic strategy and compared it with that of the spider’s venom. The results showed that the protein composition of the eggs was more complex than that of the venom and there were only a few similarities between the two materials, indicating that the eggs had the toxic components and action mechanism different from those of the venom [13]. Up to now, why the eggs of black widow spiders evolutionally acquired toxicity has not been completely elucidated. It was speculated that the toxicity of the eggs could provide a certain protection for the eggs. Although the oral feeding of *Latrodectus* eggs to mice produced no deleterious effects [8], there were experiments demonstrating that the eggs could produce toxic effect in some greedy animals. For example, Russell et al. [14] demonstrated that *Latrodectus* egg extract had deleterious effects on the web-building activity of *Araneus diadematus*. The web-building activity of the spiders receiving 3–5 g/kg body weight was abnormal. There was a significant reduction in the thread lengths and in the number of spirals. One spider receiving 1 g/kg body weight died 6 h after feeding. The relatively low toxicity of the eggs might be explained by the differences in the main roles that the proteins in eggs and venom play. The primary purpose of spider venom is to kill or paralyze preys [15] whereas the proteins in the eggs are primarily involved in the substance and energy metabolisms and their regulation during the development of the eggs into juvenile spiders [16] and their defensive role is relatively subordinate.

By using electrophysiological method, the extract of black widow spider eggs was shown to block the neuromuscular transmission in the mouse isolated phrenic nerve-hemidiaphragm preparation. This blockade appeared to be partially reversible because the contraction of diaphragm was partially recovered after washing with fresh Tyrode’s solution, which suggested that the binding of some toxic components in the egg extract to the action sites was relatively weak. After blockade, the contraction caused by direct stimulation on the diaphragm muscle remained unaffected (the trace not shown), demonstrating that the abolishment of nerve-evoked diaphragm muscle contraction was due to the blockade of neuromuscular transmission by the neurotoxic components in egg extract, not due to the decay of diaphragm muscle. In our previous work, we purified a novel neurotoxic protein with a molecular mass of 23.752 kDa, named Latroeggtoxin-I, from the eggs of black window spider by gel filtration combined with ion-exchange chromatography, and the protein was shown to be able to block the neuromuscular transmission in mouse isolated phrenic nerve-hemidiaphragm preparations completely in a reversible manner, which supported the above conclusion [17]. In addition, the mammal neurotoxic activity of the low-molecular-mass (<10 kDa) fraction was much lower than that of high-molecular-mass fraction. This suggests that the main activity of low-molecular-mass components, which include peptides, as observed by mass spectrometry, might not be mammal-neurotoxic. Our group suggests that these components might have other biological activities, such as insect toxicity or antibacterial activity, which is currently under investigation.

Ion channels are key membrane components that play important roles in controlling flow of ions across the cell membrane and regulating the excitability and functions of the cells. Many animal toxins particularly the neurotoxins exert their actions by affecting ion channels in cell membrane. Therefore, we systematically investigated the effects of the egg extract on the sodium, potassium and calcium ion channels in adult rat DRG neurons using whole-cell patch-clamp technique in order to further probe into the toxicity mechanism of the spider eggs. We first detected the effects of the extract on voltage-gated sodium channel and found that the extract could inhibit the currents of both TTX-R and TTX-S Na^+^ channels. Voltage-gated sodium channel is a transmembrane protein essential for the generation of action potentials in excitable cells [18, 19]. It has been demonstrated to be the molecular target for a number of drugs, insecticides and neurotoxins including some types of neurotoxins from spiders [20]. Our study demonstrated that the egg extract contained active components that could act on the Na^+^ channels in the cell membrane and thus affected the generation of action potentials and excitability of the cell. Furthermore, we purified a protein (named Latroeggtoxin-II) from the egg extract in our previous study. Electrophysiological experiments demonstrated that the protein can selectively inhibit TTX-R Na^+^ channel currents in rat DRG neurons, without significant effect on the TTX-S Na^+^ channel currents [21].

Besides Na^+^ channel-inhibiting components, some other components showing inhibitory activity against K^+^ channel were also found in the egg extract, which were speculated to also play some role in the toxicity of the eggs, because potassium ions flux through the K^+^ channels in cell membranes is key to numerous biological process and functions, including immunity [22], neurotransmitter release and nerve conduction [23], muscle contraction and hormone secretion [24]. Comparatively, the egg extract displayed highest inhibitory activity against the Ca^2+^ channels in the DRG neurons, judged by the inhibitory rates of the currents. When 100 μg/mL extract was applied, near 50% of the Ca^2+^ currents were inhibited, compared with less than 15% of Na^+^ and K^+^ currents being inhibited at the same concentration of the egg extract. Voltage-gated Ca^2+^ channel mediates Ca^2+^ influx into the intracellular space and control many Ca^2+^-dependent functions of the cell, including neurotransmitter release [25]. Thus, the Ca^2+^ channel-acting components in the egg extract could influence the cell excitability and many other intracellular physiological processes through suppressing the movement of calcium ions. All the results of whole-cell patch-clamp assays indicated that application of the egg extract at a concentration of 100 μg/mL could inhibit Na^+^, K^+^ and Ca^2+^ channel currents to certain degrees, suggesting that the black widow spider eggs contain multiple active components acting on the ion channels in DRG neurons. It can be speculated that these active components form the primary molecular basis of the egg toxicity.

In addition, our previous work, using proteomic strategies, demonstrated that the proteins in the eggs were involved in important cellular functions and processes including catalysis, transport and metabolism regulation, and that the proteins with enzyme activity including hydrolytic enzymes accounted for a relatively large proportion [13]. In the present study, enzyme assays showed that the egg extract possessed the activities of protease, phosphatases, acetylcholine esterase and hyalurinidase, which were in agreement with the identification results of the egg proteins with proteomic strategies [13]. These enzymes were speculated not only to participate in normal cell metabolism and its regulation, but also to be involved in the noxious action of the eggs to a certain extent. Literature survey indicated that hydrolases including proteases, lipases and phosphatases were widely found in the venoms of poisonous animals such as spiders and snakes, some of which particularly the proteases such as metalloproteases and serine proteases, had been demonstrated to have some participate in the noxious action of the venoms by degrading basement membrane molecules including laminin, entactin, type IV collagen and heparan sulfate proteoglycan [26–29].

## Conclusion

In conclusion, the eggs of black widow spiders were demonstrated to contain a mixture of proteinous compounds particularly the high-molecular-mass proteins with different types of biological activity. The neurotoxic as well as other active compounds in the eggs were speculated to form the primary molecular basis of egg toxicity different from that of the venom. In addition, the enrichment of active components in the eggs makes the eggs become a new valuable source of neurobiological tools and pharmaceutical lead compounds.

## Methods

### Reagents

Trifluoroacetic acid (TFA), α-cyano-4-hydroxycinnamic acid (CCA), acetonitrile (ACN) and dithiothreitol (DTT) were obtained from Sigma (St. Louis, MO, USA). Immobiline drystrips, ammonium persulfate, urea, agarose, glycerol, bromophenol blue, iodoacetamide (IAA), silver nitrate, 3-[(3-cholamidopropyl)- dimethylammonio]-1-propane (CHAPS), and N, N, N’, N’-tetramethylethylenediamine (TEMED) were from Amersham Pharmacia Biotech (Uppsala, Sweden). Acrylamide, Bis, Tris, glycine, SDS and SDS-PAGE protein standards were from Bio-Rad (Hercules, CA).

### Egg extract preparation

Egg extract was prepared from the eggs of about 1–2 weeks before hatching of newborns. After being washed with an insect physiological buffer (in g/L: NaCl 8.19, KCl 0.37, CaCl_2_ 0.56, MgCl_2_·6H_2_O 0.2, Glucose 0.9, HEPES 2.4, pH7.25) twice, the eggs were homogenized in a neutral buffer of weak ionic strength (10 mM PBS buffer) or ddH_2_O with a mortar and pestle. The homogenate was centrifuged at 10 000 g for 10 min at 4°C and the pellet was repeatedly homogenized and extracted twice. The supernatants were pooled and lyophilized.

### Determination of protein content and hydrolase activity

Protein content of the extract was quantitatively determined using Bradford method [30]. The determinations of protease [31], alkaline phosphatase [32], acid phosphatase [32], acetylcholine esterase [33] and hyalurinidase [34] were performed, respectively, according to the methods described previously in literature.

### Biological assay of egg extract

The extract sample was intra-abdominally injected into mice (2–6 mg/kg body weight) and cockroaches (10 μg/g body weight) to determine whether the egg extract contained components toxic to animals. Determination of LD_50_ in mice was conducted according to the methods described by Schweitz [35] and Liang et al. [36]. Briefly, the accurately weighed extract powder was dissolved in physiological saline and centrifuged at 10 000 × g for 10 min. The supernatant was used for the experiment. For LD_50_ determination, 48 mice (albino Kunming) of both sexes, weighing 20 ± 2 g, were randomly divided into eight groups, each of which consisted of six mice. Seven groups were used as experimental groups to which the extract sample solution was administrated intraperitoneally as single doses of 2.211, 2.601, 3.060, 3.600, 4.235, 4.983 and 5.862 mg/kg body weight, respectively. The eighth group was used as control and injected with physiological saline. Lethality in mice was observed during a 24 h period after injection. The LD_50_ value was determined based on the lethality in six animals at each dose level.

Furthermore, the neurotoxicity of the extract was analyzed using mouse isolated phrenic nerve-hemidiaphragm preparations according to the methods described previously [37]. Briefly, adult Kunming albino mice were killed by cervical dislocation. The phrenic nerve-hemidiaphragm preparation was isolated and placed in a small plexiglas chamber immersed in Tyrode’s solution with or without adding extract sample, continuously bubbled with a mixture of 95%O_2_ and 5%CO_2_, and maintained at 30-32°C. Electrical stimulation was applied to the phrenic nerve with a suction electrode (supramaximal voltage, 2 ms duration, square wave). The resulting twitch responses of the phrenic muscle were transformed into an electric signal by a mechanical-electric transducer. Signals were amplified and recorded with a signal process system (BL-420 S, China).

### Whole-cell patch-clamp assays

Whole-cell patch-clamp technique was employed to detect the effects of the egg extract on the ion channels in rat dorsal root ganglion (DRG) neurons. The DRGs were acutely isolated from 30-day-old Sprague–Dawley rats of either gender and the neurons prepared from the DRGs were maintained in short-term primary culture according to the methods described previously [38, 39]. The patch-clamp pipettes with a tip resistance of 2.0-3.0 MΩ were made of borosilicate glass capillary tubes. The extract-containing solutions of 10 μL volume were applied by pressure injection with a microinjector (IM-5B, Narishige, Tokyo, Jianpan) through a micropipette (20 μm in tip diameter) placed about 100 μm away from the cells under study [40]. Ion channel currents were recorded at room temperature (20–25°C). All data were presented as means ± SD, and n was used to represent the number of independent experiments and was generally ≥5.

#### Sodium currents

In order to detect the effects of egg extract on sodium currents in DRG cells, the inward sodium currents were elicited by a 50 ms step depolarization to −10 mV from a holding potential of −80 mV every 5 seconds. When the influences of the extract on the current–voltage (I-V) relationship were investigated, the sodium currents were induced by 50 ms depolarization steps to various potentials from a holding potential of −80 mV. Test potentials ranged from −80 to +70 mV in 10 mV increments. The patch-clamp pipette was filled with a solution (pH 7.2) containing (in mM) CsCl 145, MgCl_2_·6H_2_O 4, HEPES 10, EGTA 10, glucose 10, ATP 2, and the bath solution (pH 7.4) contained (in mM) NaCl 145, KCl 2.5, CaCl_2_ 1.5, MgCl_2_·6H_2_O 1.2, HEPES 10, EGTA 10, glucose 10. In view of the fact that the larger DRG neurons tend to express tetrodotoxin-sensitive (TTX-S) sodium channels whereas the smaller ones tend to express tetrodotoxin-resistent (TTX-R) sodium channels [41], the DRG neurons with diameter greater than 40 μm or smaller than 20 μm were used to detect the effects of the egg extract on TTX-S sodium currents and TTX-R sodium channels, respectively. Tetrodotoxin (0.2 μM) was added to the bath solution to separate TTX-R sodium currents from TTX-S sodium currents [42].

#### Potassium currents

For investigating the effects of the egg extract on potassium currents, the potassium currents in DRG cells were elicited by a 500 ms depolarization to +30 mV from a holding potential of −80 mV every 5 seconds. When the effects of the extract on the current–voltage (I-V) relationship were investigated, the potassium currents were induced by 50 ms depolarization steps to various potentials from a holding potential of −80 mV. Test potentials ranged from −80 to +70 mV in 10 mV increments. The suction pipette solution contained (in mM) KCl 135, KF 25, NaCl 9, MgCl_2_ 1, EGTA 1, HEPES 10 and ATP-Na_2_ 3, adjusted to pH 7.4 with 1 M KOH, and the external bath solution contained (in mM) NaCl 150, KCl 30, CaCl_2_ 5, MgCl_2_ 4, TTX 0.3, HEPES 10 and D-glucose 10, adjusted to pH 7.4 with 1 M NaOH.

#### Calcium currents

For recording calcium currents in DRG neurons in the presence and absence of the egg extract, the total calcium currents in rat DRG cells were elicited by a 150 ms depolarization to 0 mV from a holding potential of −90 mV. Low-voltage-activated (LVA) calcium channels were activated by a 100 ms step depolarization to −50 mV from a holding potential of −90 mV, whereas high-voltage-activated (HVA) calcium channels were activated by depolarization from a holding potential of −40 mV to 0 mV. The pipette internal solution contained (in mM) Cs-methane sulfonate 110, phosphocreatine 14, HEPES 10, EGTA 10, ATP-Mg 5, adjusted to pH 7.3 with CsOH, and the external bath solution contained (in mM) BaCl_2_ 10, tetraethylammonium (TEA)-Cl 125, TTX 0.3 and HEPES 10, adjusted to pH 7.4 with TEA-OH.

### MALDI TOF MS analysis

MALDI TOF mass spectrometry was used to detect the proteins and peptides with molecular masses below 10 kDa. The low-molecular-mass fraction was prepared by ultrafiltrating the egg extract with a centrifugal filter (10 000 MWCO, Millipore). Mass spectrometric analysis was performed on an ultraflex TOF/TOF mass spectrometer (Bruker Daltonics Inc.). Acquisition operation mode was linear. Sample solution was mixed with the saturated α-cyano-4-hydroxycinnamic acid solution (prepared with 50% ACN containing 0.1% TFA) at a ratio of 1:1 and 1 μL of the mixture solution (about 1 μg proteins/peptides) was applied onto the sample carrier for the analysis.

### SDS-PAGE of extract and venom

SDS-PAGE of the egg extract and venom was performed according to the method of Laemmli [43] under denaturing conditions on a 4.8% stacking gel and an 11.5% separation gel. Aliquots of lyophilized extract and venom (each containing 100 μg proteins) were separately dissolved in 30 μL of sample buffer (50 mM Tris–HCl, pH 6.8, 65 mM DTT, 0.5 mM phenylmethylsulfonyl fluoride (PMSF), 2%SDS, and a trace of bromophenol blue) and boiled for 3 min. The sample solutions were centrifuged at 10 000 × g for 15 min and the supernatants were loaded into the parallel gel wells. The SDS-PAGE was run at 25 mA on the stacking gel and at 45 mA on the separating gel. After carrying out the electrophoresis, the separated proteins were visualized by Coomassie brilliant blue G-250 staining. A prestained protein ladder (from Bio-Rad) was used as standard molecular mass markers.

### 2D-PAGE of extract

The lyophilized egg extract was separated with two-dimensional gel electrophoresis (2D-PAGE) according to the method previously described [44] to gain more detail information on the large protein components in the eggs. 500 μg of the extract powder was dissolved in about 350 μL of rehydration solution (8 M urea, 4% (w/v) CHAPS, 65 mM DTT, 0.5% (v/v) IPG buffer, 0.5% pharmalyte, a trace of Bromophenol Blue). The mixture solution was clarified by centrifugation at 10 000 × g for 10 min. Commercial 18 cm IPG strip (Bio-Rad) with a linear range of pH3-10 was rehydrated overnight with the sample solution. Isoelectric focusing was performed in a Bio-Rad Protean isoelectric focusing unit according to the method described by the manufacturer. The conditions for isoelectric focusing were as follows: 30 V for 14 h; 500 V for 1 h; 1000 V for 1 h; 8000 V for up to 32000 Vh. Before running the second dimension, the IPG strip was placed in a tray and the egg proteins in the strips were reduced and alkylated by sequential incubation in equilibration solution A (0.05 M Tris–HCl, pH6.8, 8 M urea, 30% glycerol, 1% SDS and 0.2% DTT) for 15 min, and in equilibration solution B (0.05 M Tris–HCl, pH6.8, 8 M urea, 30% glycerol, 1% SDS, 3% IAA and a trace of bromophenol blue) for another 15 min. For the second electrophoresis separation, the strip was embedded on top of the 2D gel and covered with agarose. Second–dimensional SDS-PAGE was performed on 5% polyacrylamide stacking gel (25 mA per gel) and 12% polyacrylamide separating gel (50 mA per gel) until the dye front reached near the bottom of the gel at a temperature of 10°C. The separated egg proteins in the gel were visualized by Coomassie brilliant blue G-250 staining.

All procedures conformed to the Guidelines of the National Institutes of Health Guide for the Care and Use Laboratory Animals. The present study was approved by the Ethics Committee on the Use and Care of Animals of the Hunan Province, P. R. China.

## Abbreviations

MALDI TOF MS: Matrix-assisted laser desorption/ionization time-of-flight mass spectrometry
SDS-PAGE: Sodium dodecyl sulfate- polyacrylamide gel electrophoresis
2D-PAGE: Two-dimensional polyacrylamide gel electrophoresis
DRG: Dorsal root ganglion.
